# Supplementary figures and images for: Architecture of the chikungunya virus replication organelle
Source: eLife. 2022 Oct 19;11:e83042. doi: 10.7554/eLife.83042 (PMC9633065; doi:10.7554/eLife.83042)

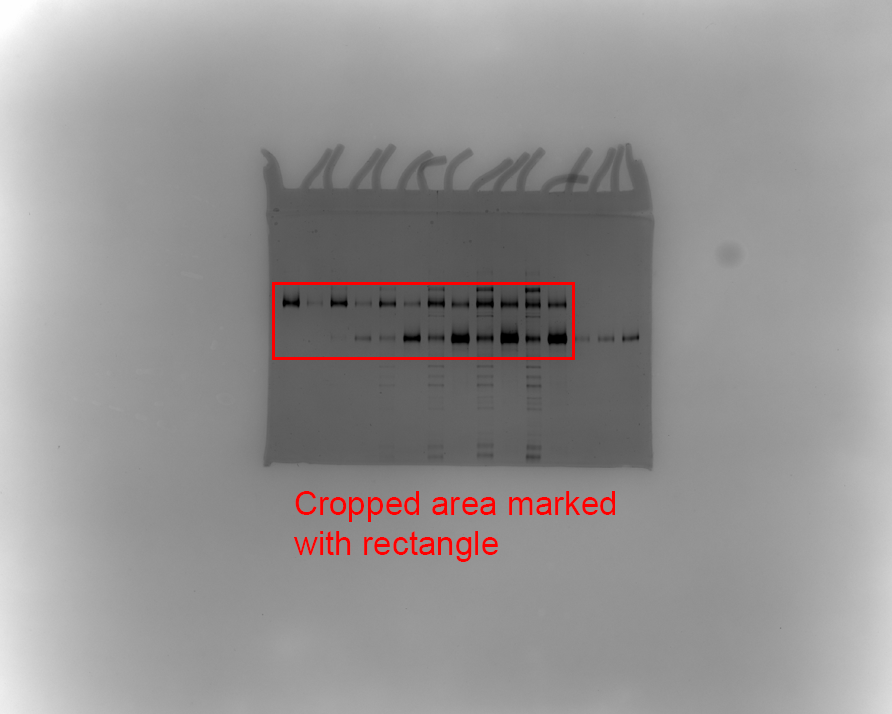

Supplement: Figure 3—source data 1. [file elife-83042-fig3-data1.zip › Figure 3 - source data 1/Figure 3F marked.tif]

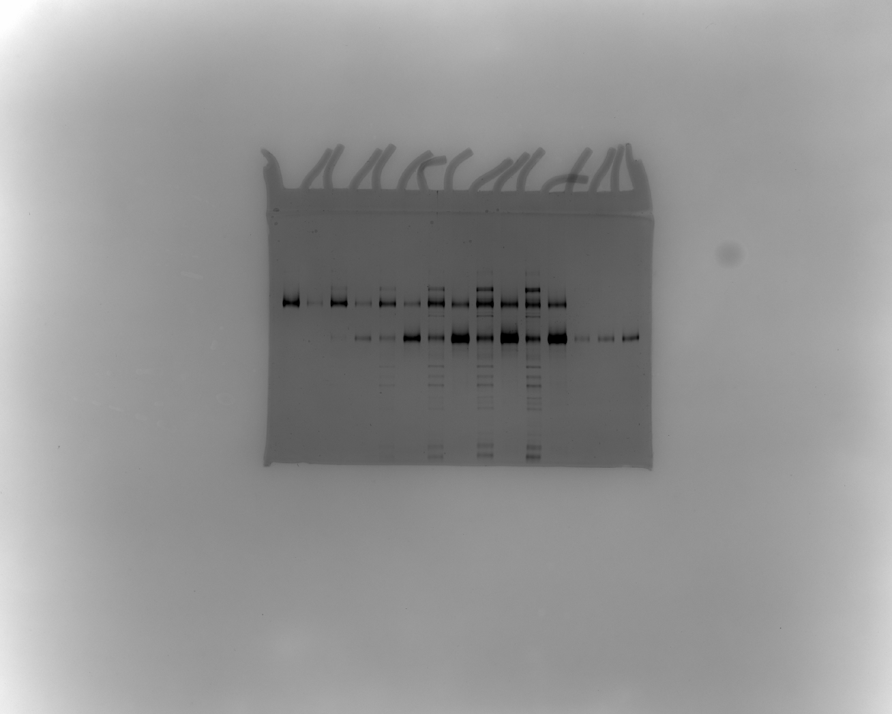

Supplement: Figure 3—source data 1. [file elife-83042-fig3-data1.zip › Figure 3 - source data 1/Figure 3F.tif]

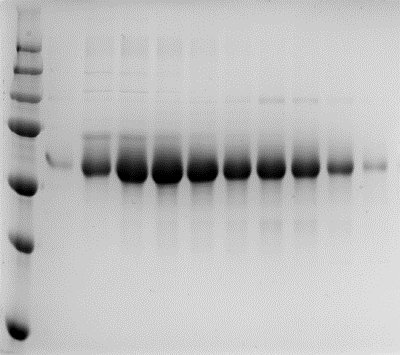

Supplement: Figure 3—figure supplement 1—source data 1. [file elife-83042-fig3-figsupp1-data1.zip › Figure 3 - figure supplement 1 - source data 1/Figure 3 - figure supplement 1B.jpg]

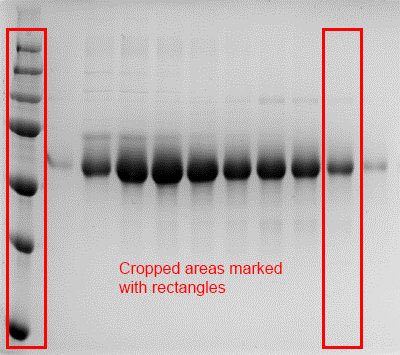

Supplement: Figure 3—figure supplement 1—source data 1. [file elife-83042-fig3-figsupp1-data1.zip › Figure 3 - figure supplement 1 - source data 1/Figure 3 - figure supplement 1B_marked.jpg]

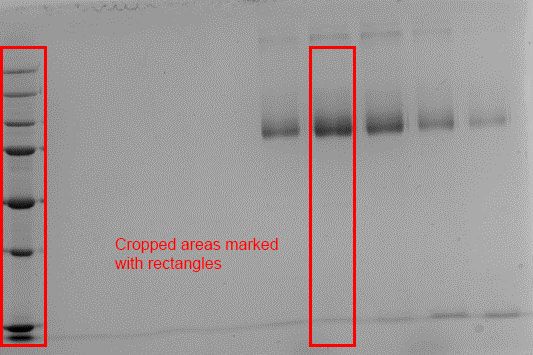

Supplement: Figure 3—figure supplement 1—source data 1. [file elife-83042-fig3-figsupp1-data1.zip › Figure 3 - figure supplement 1 - source data 1/Figure 3 - figure supplement 1E marked.jpg]

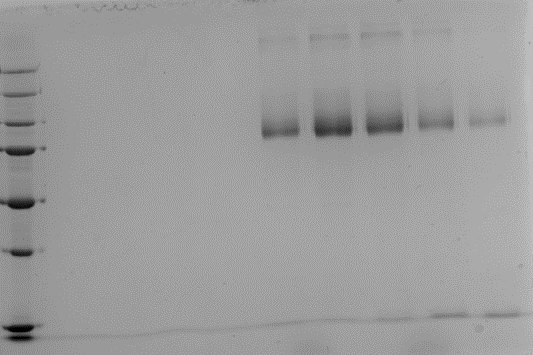

Supplement: Figure 3—figure supplement 1—source data 1. [file elife-83042-fig3-figsupp1-data1.zip › Figure 3 - figure supplement 1 - source data 1/Figure 3 - figure supplement 1E.jpg]

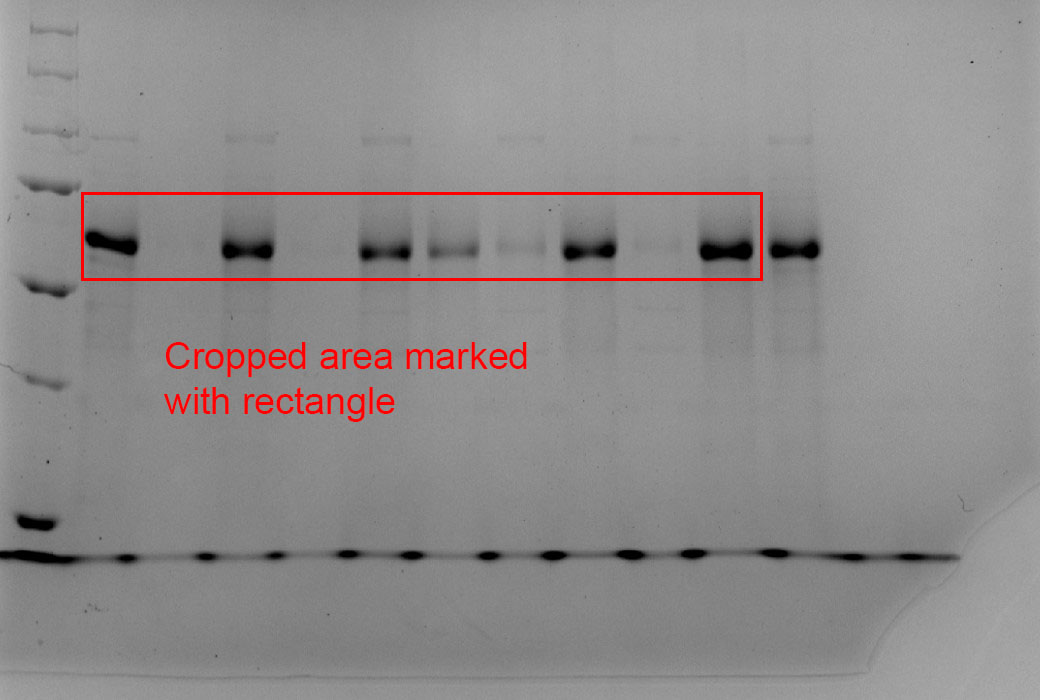

Supplement: Figure 3—figure supplement 2—source data 1. [file elife-83042-fig3-figsupp2-data1.zip › Figure 3 - figure supplement 2 - source data 1/Figure 3 - figure supplement 2A marked.jpg]

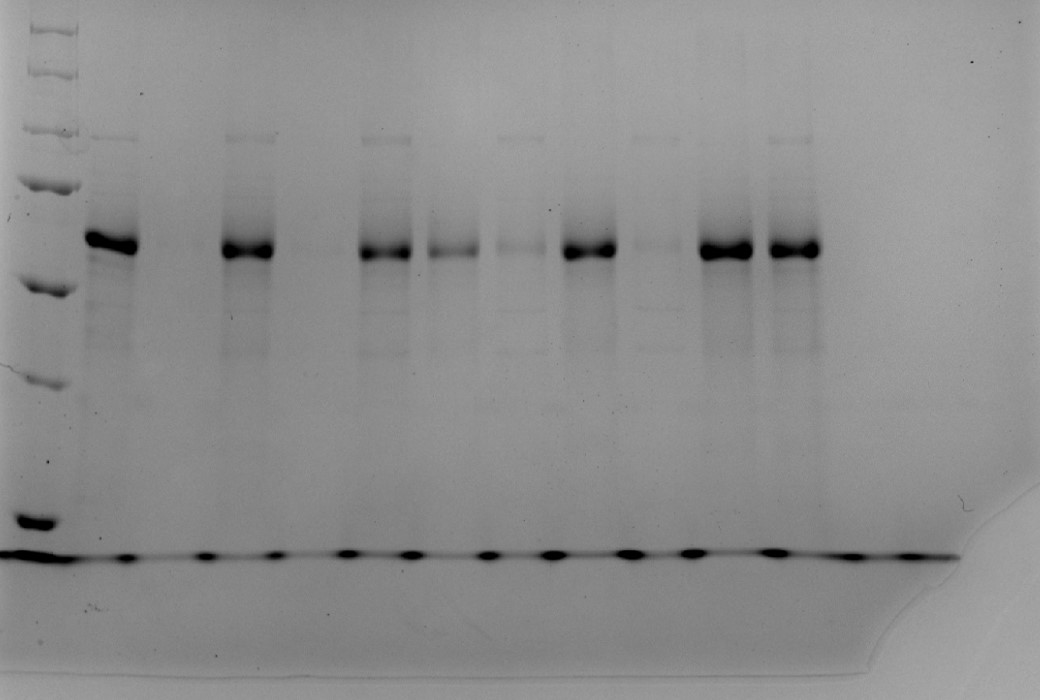

Supplement: Figure 3—figure supplement 2—source data 1. [file elife-83042-fig3-figsupp2-data1.zip › Figure 3 - figure supplement 2 - source data 1/Figure 3 - figure supplement 2A.jpg]

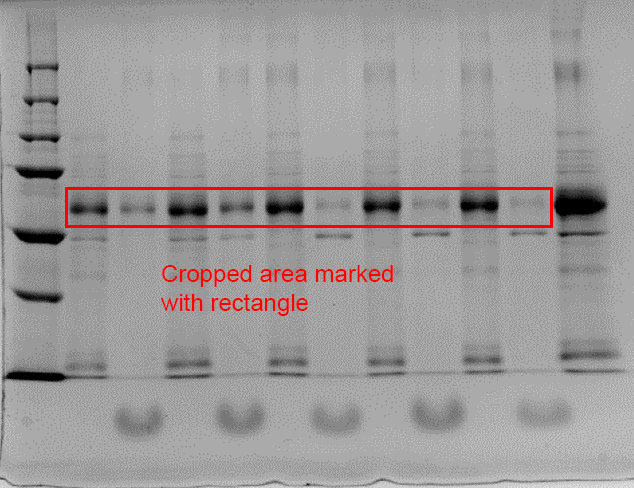

Supplement: Figure 3—figure supplement 2—source data 1. [file elife-83042-fig3-figsupp2-data1.zip › Figure 3 - figure supplement 2 - source data 1/Figure 3 - figure supplement 2B marked.jpg]

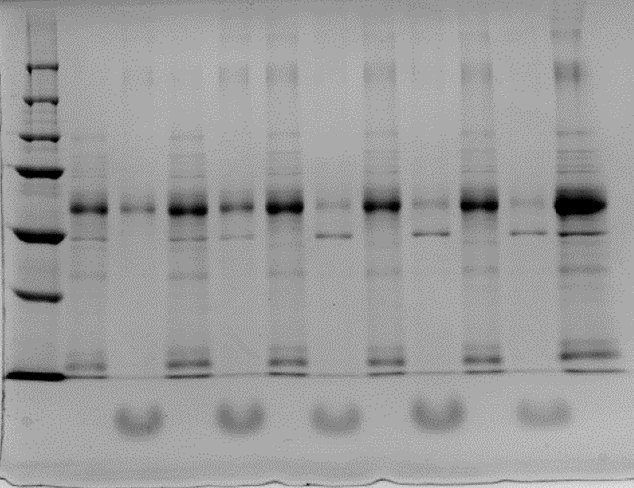

Supplement: Figure 3—figure supplement 2—source data 1. [file elife-83042-fig3-figsupp2-data1.zip › Figure 3 - figure supplement 2 - source data 1/Figure 3 - figure supplement 2B.jpg]

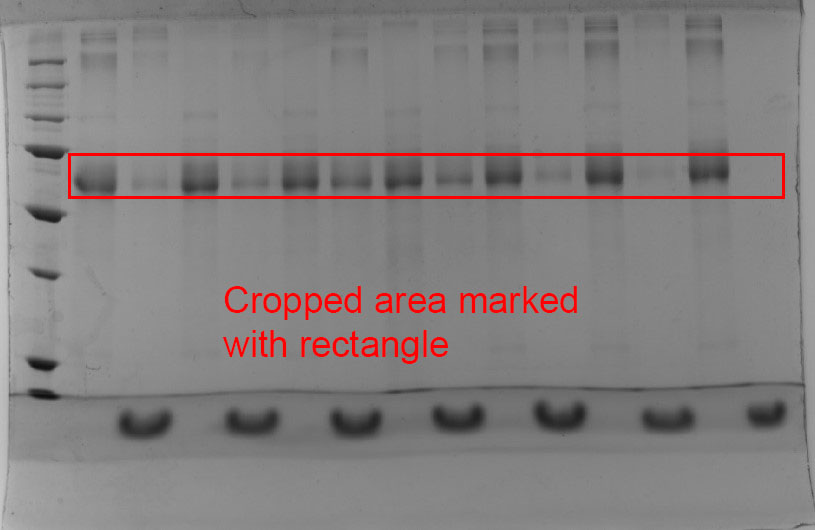

Supplement: Figure 3—figure supplement 2—source data 1. [file elife-83042-fig3-figsupp2-data1.zip › Figure 3 - figure supplement 2 - source data 1/Figure 3 - figure supplement 2C marked.jpg]

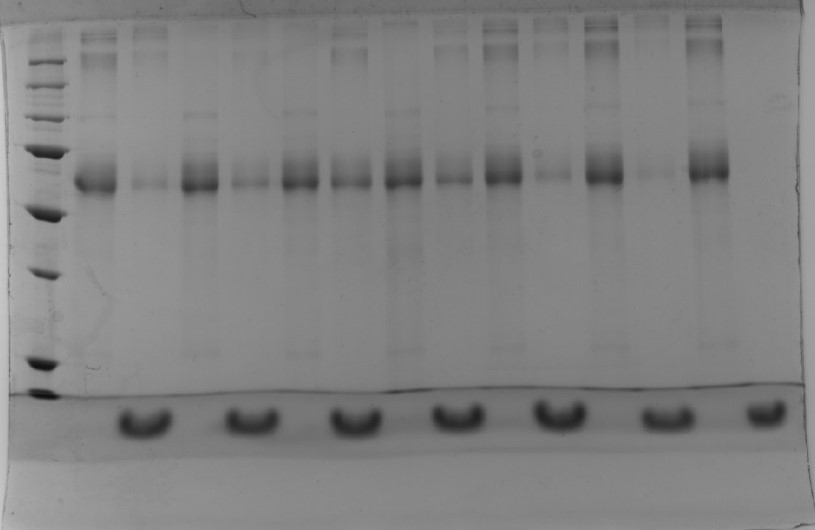

Supplement: Figure 3—figure supplement 2—source data 1. [file elife-83042-fig3-figsupp2-data1.zip › Figure 3 - figure supplement 2 - source data 1/Figure 3 - figure supplement 2C.jpg]
